# Cytokeratin 18 can help predict liver fibrosis in HCV infected patients with type 2 diabetes mellitus

**DOI:** 10.1186/s12876-021-01963-7

**Published:** 2021-10-20

**Authors:** Fang Li, Feifei Lei, Chengcai Wen, Qiu Ge, Liyao Zhu

**Affiliations:** 1 Department of Infectious Diseases, Institute of Hepatic Diseases, Renmin Hospital, Hubei University of Medicine, Shiyan, China; 2 Huai’an Second People’s Hospital, The Affiliated Huai’an Hospital of Xuzhou Medical University, Huai’an, China; 3 Outpatient Department, The Second People’s Hospital of Huai’an, The Affiliated Huai’an Hospital of Xuzhou Medical University, Huai’an, China; 4 Department of Hepatology, The Fourth People’s Hospital of Huai’an, Jiangsu, China

**Keywords:** Hepatitis C virus (HCV), Liver fibrosis, Type 2 diabetes mellitus (T2DM), Cytokeratin 18 (CK18), Predictive values

## Abstract

**Background:**

To investigate the predictive values of cytokeratin 18 for liver fibrosis in hepatitis C virus (HCV) infected patients with type 2 diabetes mellitus (T2DM).

**Methods:**

252 HCV-infected patients with T2DM between January 2012 and August 2017 were retrospectively reviewed. Pearson/spearman correlation analysis was used to detect the correlation in the entire cohort. Multivariate linear regression was used to identify independent predictors and logistic regression was for establishing models. Combination models that incorporated CK18 and other methods (i.e. transient elastography, aspartate transaminase-to-platelet ratio index (APRI) and fibrosis-4 index (FIB-4)] were developed in a training cohort of 132 patients. Performance of models was evaluated through discrimination ability and clinical benefits. An internal validation was conducted in 120 consecutive patients.

**Results:**

CK18 was found significantly associated with fibrosis scores (*r* = 0.452, *P* < .001). CK18 and albumin were confirmed as independent predictors for fibrosis. For predicting significant fibrosis in the validation cohort, the observed AUC values of APRI + CK18 (AUC 0.83) and FIB-4 + CK18 (AUC 0.84) were higher than those of APRI (AUC 0.61) and FIB-4 (AUC 0.65). For predicting advanced fibrosis and cirrhosis, the AUC values of FIB-4 + CK18 (AUC 0.74 and 0.77, respectively) were significantly higher than those of FIB-4 (AUC 0.61 of both). Decision curve analysis confirmed the more clinical benefits can be provided by being combined with CK18.

**Conclusions:**

CK18 is an independent predictor of liver fibrosis for HCV-infected patients with T2DM. Noninvasive methods incorporate CK18 and other biomarker indices can have better performance for diagnosing fibrosis and help clinical decision-making.

## Introduction

As estimated by world health organization (WHO) in 2015, 71 million patients were infected with hepatitis C virus (HCV) and 399,000 died because of cirrhosis or hepatocellular carcinoma cause by HCV in the worldwide [1]. About 422 million people worldwide have diabetes mellitus, which have become one of the leading causes of death [2, 3]. Both HCV and diabetes mellitus contribute to the global burden of disease.

Chronic hepatitis C (CHC) has been found associated with a four-fold increased risk of insulin resistance and type 2 diabetes mellitus (T2DM) [4]. Meanwhile, hyperglycemia and insulin which can stimulate hepatic stellate cells mitogenesis and collagen synthesis, are key factors in the progression of fibrosis [5, 6]. A previous study [7] conducted by Wieckowska et al. showed that cytokeratin 18 (CK18) is at high levels and positively correlated with liver fibrosis stage in patients with non-alcoholic fatty liver disease (NAFLD). Moreover, CK18 has been proposed for direct measures of inflammation in NAFLD patients [8, 9].

However, only a few studies investigated the correlations about CK18 in patients infected with HCV [10, 11], and limited data confirmed the strong relationship between serum CK18 and HCV-related fibrosis. Sanyal et al. [10] also found CK18 associated with insulins in CHC complicated with T2DM. Additionally, there have been many developed noninvasive serum biomarkers for predicting liver fibrosis in CHC patients without any other metabolic diseases [12], but no marker was specially developed for CHC-T2DM patients. The aim of this study was to investigate the association between the level of CK18 and liver fibrosis and evaluate the predictive value of CK18 in CHC patients with T2DM.

## Materials and methods

This retrospective study was approved by the institutional review board of the Fourth People’s Hospital of Huai’an. The requirement for written informed consent was waived by the institutional review board of the Fourth People’s Hospital of Huai’an due to its retrospective nature. All methods were performed in accordance with the 1975 Helsinki declaration and its later amendments.

### Patients

**Fig. 1:**
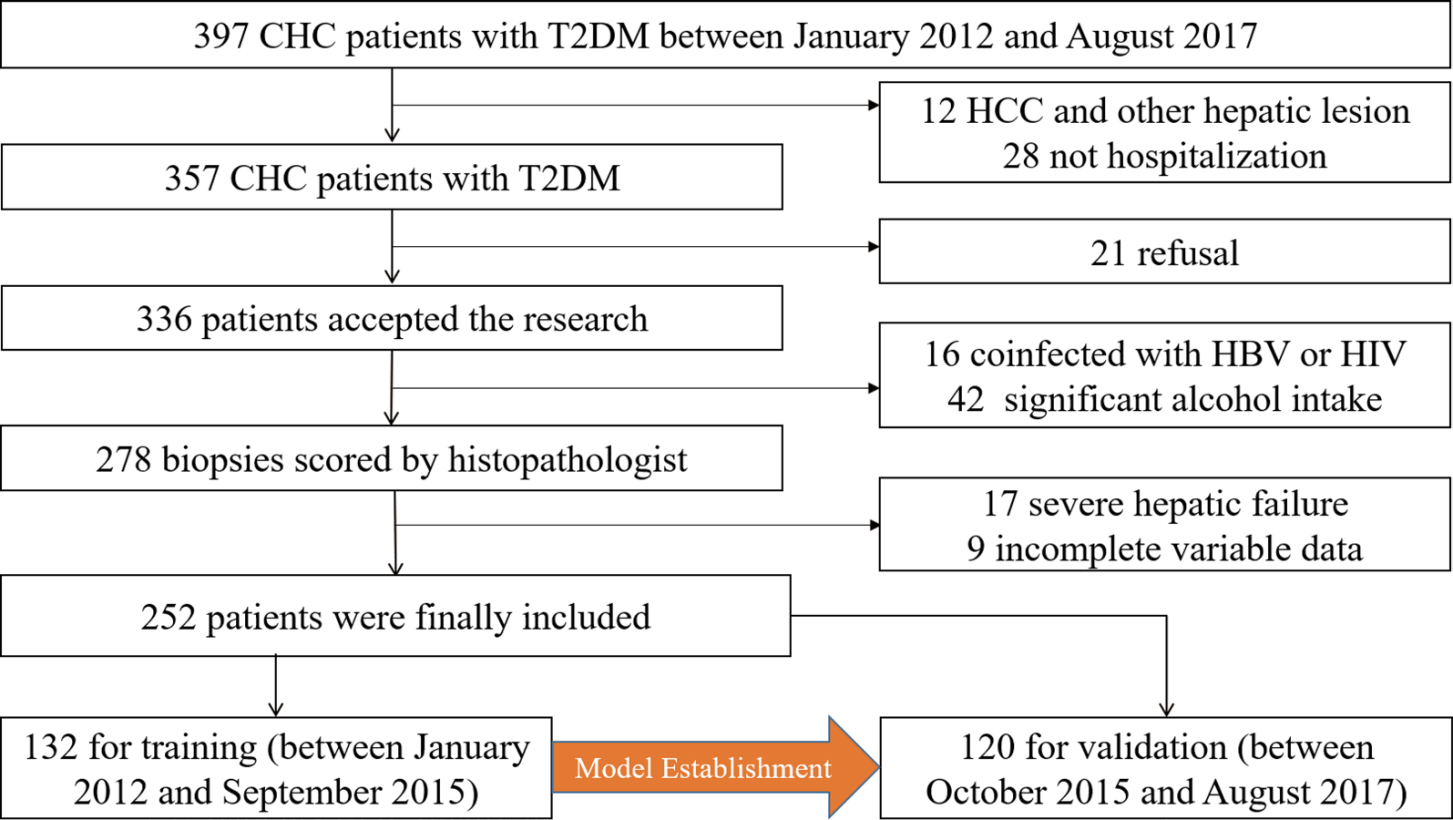
Patient selection flow chart. CHC, chronic hepatitis C; HBV, hepatitis B virus; HCC, hepatocellular carcinoma; HIV, human immunodeficiency virus

The flow chart is shown in Fig. 1. 397 patients diagnosed with CHC and T2DM, who were admitted to the Fourth People’s Hospital of Huai’an between January 2012 and August 2017 were retrospectively reviewed. The inclusion criteria were shown as follows: (a) diagnosed with CHC (positive anti-HCV antibodies for over 6 months and HCV RNA > 1.0 × 10^3^ IU/ml); (b) clinically determined diabetes; (c) treat-naïve before hospitalization in this hospital. The exclusion criteria were: (a) focal hepatic lesion (i.e. tumor, hepatic tuberculosis and any other); (b) coinfected with other virus such as hepatitis B virus (HBV), hepatitis D virus (HDV) and human immunodeficiency virus (HIV); (c) significant alcohol intake (> 20 g/day); (d) severe hepatic failure (jaundice and ascites or transaminases level more than 10 times the upper limit of normal); (e) inadequate clinical data. Patients were allocated to the training and validation cohort according to the time of hospitalization (Training: January 2012-September 2015; Validation: October 2015-August 2017).

Clinical data included age, sex, body mass index (BMI), plate count (PLT), prothrombin time (PT), international normalized ratio (INR), fasting blood glucose (FBG), fasting insulin (FINS), alanine aminotransferase (ALT), aspartate aminotransferase (AST), alkaline phosphatase (ALP), glutamyl transpeptidase (GGT), lactate dehydrogenase (LDH), total cholesterol (TC), triacylglycerol (TG), high density lipoprotein cholesterol (HDL-C) and low density lipoprotein cholesterol (LDL-C) at the first diagnosis of CHB and T2DM. Homeostatic model assessment for insulin resistance (HOMA-IR) score was calculated using the formula: HOMA-IR = FINS (mIU/L) × FBG (mmol/L)/22.5.

### Liver biopsy

Liver biopsy (LB) was performed by experienced ultrasonologists and liver samples were formalin-fixed and paraffin-embedded for analysis. Two senior pathologists, who were blinded to the clinical information, determined final histological results in consensus. Liver fibrosis was scored according to the Metavir system [13]. F ≥ 2 was regarded as significant fibrosis, F ≥ 3 as advanced fibrosis and F4 as cirrhosis.

### Transient elastography (Fibroscan)

United Fibroscan devices (FS402, Echosens, France) were used for measuring liver stiffness according to the manufacturer’s protocol [14]. The transient elastography (TE) results were expressed in kilopascal (kPa) and the final measuring result was the median value of 10 successful acquisitions.

### Serum index calculation

Because of simple formulas and easily available parameters, aspartate transaminase (AST)-to-platelet ratio index (APRI) and the fibrosis-4 (FIB-4) were frequently used [15, 16]. Formulas were as follows:

$${\text{APRI }} = \frac{{\left( {\text{AST(IU/L) / ULN}} \right)\, \times \, 100 }}{{{\text{Platelet}}\,{\text{count}} \left( {10^{9} {\text{/L}}} \right)}}$$

$${\text{FIB - 4 }} = \frac{{{\text{age}}\,({\text{years}}) \times {\text{AST}}\,\left( {\text{IU/L}} \right) }}{{{\text{Platelet}}\,{\text{count}} \left( {10^{9} {\text{/L}}} \right) \times {\text{ALT}}\,\left( {\text{IU/L}} \right)^{ \wedge } 1/2}}$$

### Measurement of CK18 in patients

During liver biopsy, a blood sample was obtained from each patient and processed to plasma (stored at − 80 °C). CK18 was quantitatively measured using the M30-Apoptisense ELISA kit (PEVIVA; Alexis, Grünwald, Germany) from the plasma. All measurements were performed in triplicate and the absorbance was determined using a microplate reader (Molecular Devices M2, Sunnyvale, CA).

### Sample size calculation

The sample size was calculated according to 10 events per variable (EPV). The calculation was done based on the assumption that the possible significant variables including ALT, AST, GGT, PLT and CK18 according to previous studies about non-invasive tests for evaluation of liver disease severity and prognosis. A total of 5 variables were considered as possibly significant. Then, the estimated minimum sample size was 100.

### Statistical analysis

Continuous data were presented as means with standard deviations or medians with percentiles (25th and 75th), and the comparisons between two groups were analyzed by the Mann–Whitney *U* test. Count data were presented as percentages and the comparison was performed by the χ^2^ test. Pearson and spearman correlation analysis were used for categorical and continuous data, respectively. Forward conditional linear multivariate analysis was used to capture independent correlations for multivariate data. All combination models were established using logistic regression in the training cohort. The optimal cutoff value was determined due to the receiver operating characteristic (ROC) curve by maximizing the Youden index (sensitivity + specificity − 1). The validation cohort tested the model using the formula and optimal cutoff values derived from the primary cohort. The discrimination ability of models was quantified by the ROC curve and area under the curve (AUC) value. Delong test was used to compare AUC values. Decision curve analysis (DCA) was used to calculate the net benefit from the models at different threshold probabilities. A two-sided *P* value less than 0.05 was considered statistically significant.

## Results

### Main characteristics for CHC-T2DM patients

In total, 252 patients between January 2012 and August 2017 were finally included: 132 (between January 2012 and September 2015) were allocated for training and 120 (between October 2015 and August 2017) for internal validation. As summarized in Table 1, no differences were observed in CK18 and other clinical-pathological characteristics between the training and validation cohorts. The overall rate of significant fibrosis, advanced fibrosis and cirrhosis was 77.0% (194 of 252), 58.7% (148 of 252) and 38.5% (97 of 252).

**Table 1:** Main characteristics

Parameter	Training (n = 132)	Validation (n = 120)	*P* value
Sex			.614
No. of men	72 (54.5)	61 (50.8)	
No. of women	60 (45.5)	59 (49.2)	
Age			.703
< 60 (years)	76 (57.6)	72 (60.0)	
≥ 60 (years)	56 (42.4)	48 (40.0)	
BMI	23.86 ± 3.06	23.67 ± 2.88	.618
Laboratory findings^a^			
WBC (10^9^/L)	5.45 ± 2.15	5.20 ± 2.04	.354
FBG (mmol/L)	8.6 ± 4.2	9.3 ± 5.4	.250
FINS (mIU/L)	14.86 ± 10.37	16.33 ± 11.49	.287
AST (IU/mL)	57.59 ± 68.80	69.88 ± 121.65	.319
ALT (IU/mL)	86.45 ± 145.88	87.22 ± 169.00	.969
GGT (IU/mL)	92.16 ± 118.81	77.40 ± 106.36	.302
ALP (IU/mL)	98.39 ± 60.50	87.78 ± 67.94	.191
Platelet count (10^9^/L)	151.96 ± 59.70	148.97 ± 68.49	.711
Cholesterol (mmol/L)	3.96 ± 1.00	4.04 ± 0.92	.527
TG (mmol/L)	1.80 ± 1.21	1.60 ± 1.05	.106
INR	1.17 ± 0.18	1.17 ± 0.15	.774
APRI	1.17 ± 1.58	1.52 ± 3.31	.275
FIB-4	2.16 ± 1.67	2.69 ± 3.42	.117
Histologic activity grade			.271
A0	27 (20.5)	23 (19.2)	.875
A1	49 (37.1)	57 (47.5)	.099
A2	40 (30.3)	32 (26.7)	.577
A3	16 (12.1)	8 (6.7)	.197
Histologic fibrosis stage			.787
F0	11 (8.3)	10 (8.3)	> .99
F1	16 (12.1)	21 (17.5)	.285
F2	25 (18.9)	21 (17.5)	.871
F3	29 (22.0)	22 (18.3)	.531
F4	51 (38.6)	46 (38.3)	.961
TE (kPa)	11.13 ± 5.21	11.05 ± 5.87	.914
CK18 (ng/L)	105.41 ± 28.33	103.24 ± 26.67	.533

Except where indicated, data are numbers of patients, with percentages in parentheses

ALT, alanine transferase; ALP, alkaline phosphatase; APRI, aspartate transaminase-to-platelet ratio; AST, aspartate transaminase; BMI, body mass index; CK18, cytokeratin 18; FBG, fasting blood glucose; FINS, fasting insulin; FIB-4, fibrosis-4 index; GGT, γ-glutamyl transferase; INR, international normalized ratio; TE, transient elastography; TG, triglyceride; WBC, white blood cell

^a^Data are presented as means ± standard deviations

### Correlation between CK18 and Fibrosis score in the entire cohort

**Fig. 2:**
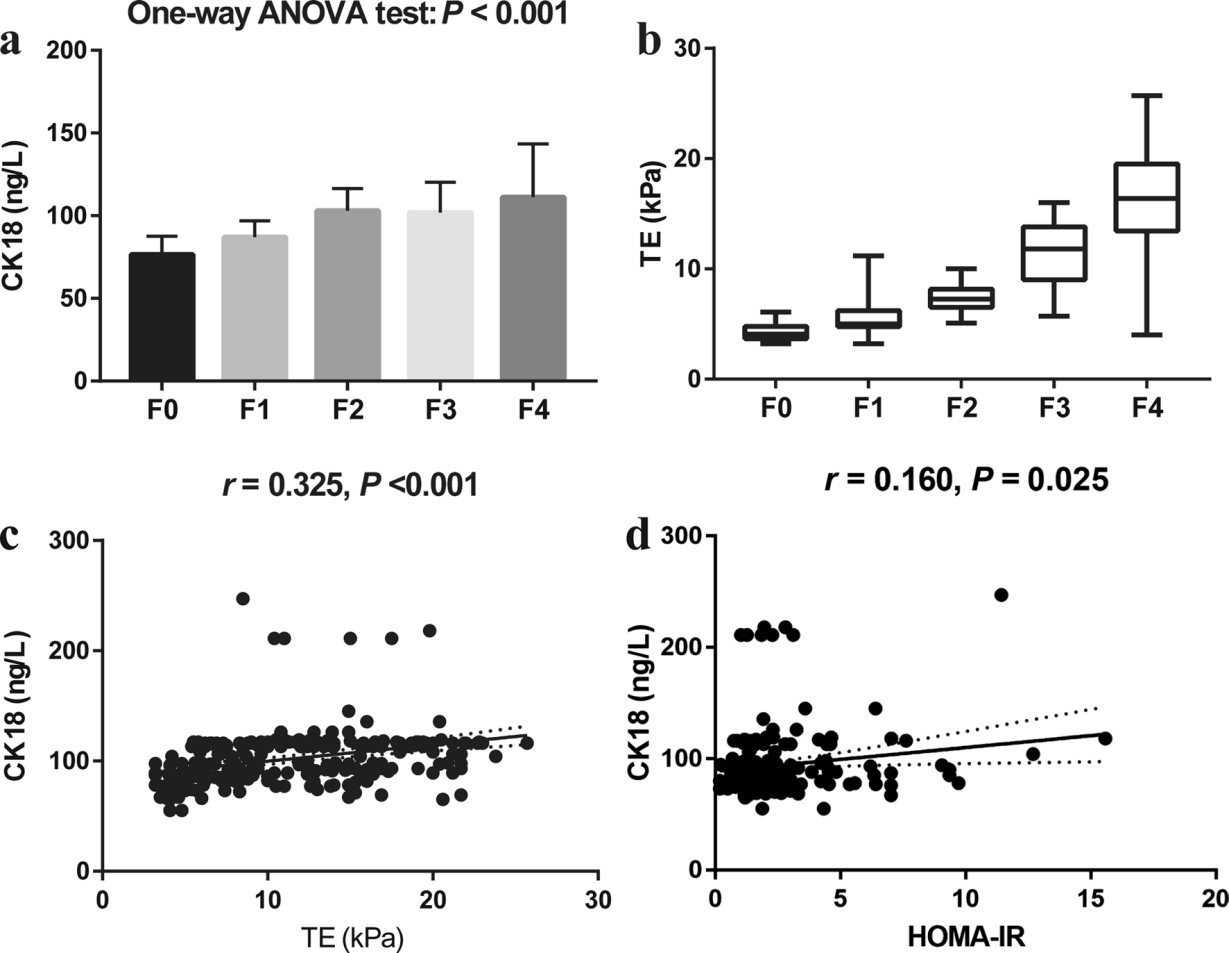
Correlation analyses. **a** One-way ANOVA test of CK18 due to fibrosis stages; **b** Boxplots of TE due to fibrosis stages; **c** Pearson correlation analysis of CK18 and TE; **d** Pearson correlation analysis of CK18 and HOMA-IR. CK18, cytokeratin 18; HOMA-IR, homeostasis model assessment of insulin resistance; TE, transient elastography

As is shown in Fig. 2, the level of CK18 was found significantly positively associated with fibrosis scores (Spearman correlation analysis, *r* = 0.452, *P* < 0.001). The CK18 of patients in F0, F1, F2, F3 and F4 fibrosis group was 76.6 ± 10.9 (ng/L), 87.1 ± 9.7 (ng/L), 103.0 ± 13.3 (ng/L), 101.8 ± 18.4 (ng/L) and 111.3 ± 32.2 (ng/L), respectively. TE had the ability of reflecting the actual progression of liver fibrosis (Pearson correlation analysis, *r* = 0.855, *P* < 0.001). The significant positive correlation between CK18 and TE (Pearson correlation analysis, *r* = 0.325, *P* < 0.001) also provided additional evidence of the relation between CK18 and histologic fibrosis score. In addition, the level CK18 is positively associated with HOMA-IR (Pearson correlation analysis, *r* = 0.160, *P* = 0.025).

### Independent predictors for liver fibrosis

In initial spearman correlation analysis (Table 2), ALB, WBC, PLT, PT, INR, TE and CK18 were observed significantly related to fibrosis stage (*P* < 0.005 for all). Through the forward conditional linear multivariable analysis, ALB, CK18 and TE were confirmed as independent predictors for the prediction of fibrosis stage (*P* = 0.008, < 0.001, < 0.001, respectively). As summarized in Table 3, the cutoff values of isolated CK18 for the prediction of significant fibrosis, advanced fibrosis and cirrhosis were 90.5 (ng/L), 90.5 (ng/L) and 104 (ng/L), respectively.

**Table 2:** Clinical characteristics of the training cohort related to fibrosis stage

Variables	Spearman correlation analysis		Multivariable analysis	
	*r*^2^ value	*P* value	*b* coefficient	*P* value
Age (years)	0.018	.124	NA	NA
Sex (male, female)	0.023	.085	NA	NA
BMI	0.001	.743	NA	NA
Child–Pugh (A, B + C)	0.003	.142	NA	NA
ALT (U/L)	0.001	.761	NA	NA
AST (U/L)	0.021	.146	NA	NA
ALP (U/L)	0.018	.124	NA	NA
GGT (U/L)	0.024	.076	NA	NA
TB (µmol/L)	0.019	.117	NA	NA
CB (µmol/L)	0.031	.102	NA	NA
ALB (g/L)	0.085	.001	− 0.030	.008
GLOB (g/L)	0.013	.064	NA	NA
CRP (mg/L)	0.009	.088	NA	NA
WBC (10^9^/L)	0.038	.024	NA	.916
PLT (10^9^/L)	0.189	< .001	NA	.279
PT (s)	0.092	< .001	NA	.304
INR	0.121	< .001	NA	.198
Cholesterol (mmol/L)	0.000	.914	NA	NA
TG (mmol/L)	0.026	.065	NA	NA
HDL-C	0.005	.402	NA	NA
LDL-C	0.002	.688	NA	NA
FBG (mmol/L)	0.016	.153	NA	NA
FINS (mIU/L)	0.015	.048	NA	.234
IR	0.012	.026	NA	.131
TE (kPa)	0.736	< .001	0.189	< .001
CK18 (ng/L)	0.211	< .001	0.009	< .001

*b* coefficients are from multivariable linear regression

ALB, albumin; ALP, alkaline phosphatase; ALT, alanine aminotransferase; AST, aspartate aminotransferase; BMI, body mass index; CK18, cytokeratin 18; CRP, C reactive protein; FBG, fasting blood glucose; FINS, fasting insulin; GGT, γ-glutamyl transferase; GLOB, globulin; HDL-C, high density lipoprotein cholesterol; INR, international normalized ratio; IR, insulin resistance; LDL-C, low density lipoprotein cholesterol; TE, transient elastography; TG, triglyceride; PLT, blood platelet; PT, prothrombin time; TB, serum total bilirubin; WBC, white blood cell

**Table 3:** The optimal cutoff values of CK18 for the prediction of significant fibrosis, advanced fibrosis and cirrhosis

CK18	Significant fibrosis (F0-1 vs F2-4)	Advanced fibrosis (F0-2 vs F3-4)	Cirrhosis (F0-3 vs F4)
AUROC (95%CI)	0.86 (0.80, 0.93)	0.75 (0.66, 0.83)	0.84 (0.79, 0.89)
Cutoff values (ng/L)	90.5	90.5	104
Sensitivity/specificity (%)	82/81	84/54	77/83
Correctly classified (%)	82	72	78
PPV/NPV (%)	95/54	74/68	94/52
Positive/negative LR	4.42/0.22	1.81/0.30	4.45/0.28

Numbers in parentheses are the 95% confidence interval

AUROC, area under the receiver operating characteristic; CK18, cytokeratin 18; LR, likelihood ratio; NPV, negative predictive value; PPV, positive predictive value

### Predictive values of CK18

According to the cutoff values, the input values of CK18 were set as ordered categorical data (0, ≤ 90.5 ng/L; 1, > 90.5 & ≤ 104 ng/L; 2, > 104 ng/L). The formula of combination models for predicting significant fibrosis, advanced fibrosis and cirrhosis are developed from the training cohort and summarized in Table 4.

**Table 4:** The formula of established prediction methods for significant fibrosis, advanced fibrosis and cirrhosis

	Significant fibrosis (F0-1 vs F2-4)	Advanced fibrosis (F0-2 vs F3-4)	Cirrhosis (F0-3 vs F4)
TE + CK18	0.945*TE + 1.252*CK18 − 6.765	1.003*TE + 0.335*CK18 − 9.157	0.465*TE + 0.576*CK18 − 6.903
APRI + CK18	0.399*APRI + 1.858*CK18 − 0.680	− 0.022*APRI + 1.164*CK18 − 0.987	− 0.006*APRI + 1.175*CK18 − 2.137
FIB-4 + CK18	0.331*FIB-4 + 1.823*CK18 − 0.894	0.227*FIB-4 + 1.133*CK18 − 1.452	0.442*FIB-4 + 1.226*CK18 − 3.228

Numbers in parentheses are the 95% confidence interval

AUROC, area under the receiver operating characteristic; APRI, aspartate transaminase-to-platelet ratio; CK18, cytokeratin 18; FIB-4, fibrosis-4 index; TE, transient elastography

**Fig. 3:**
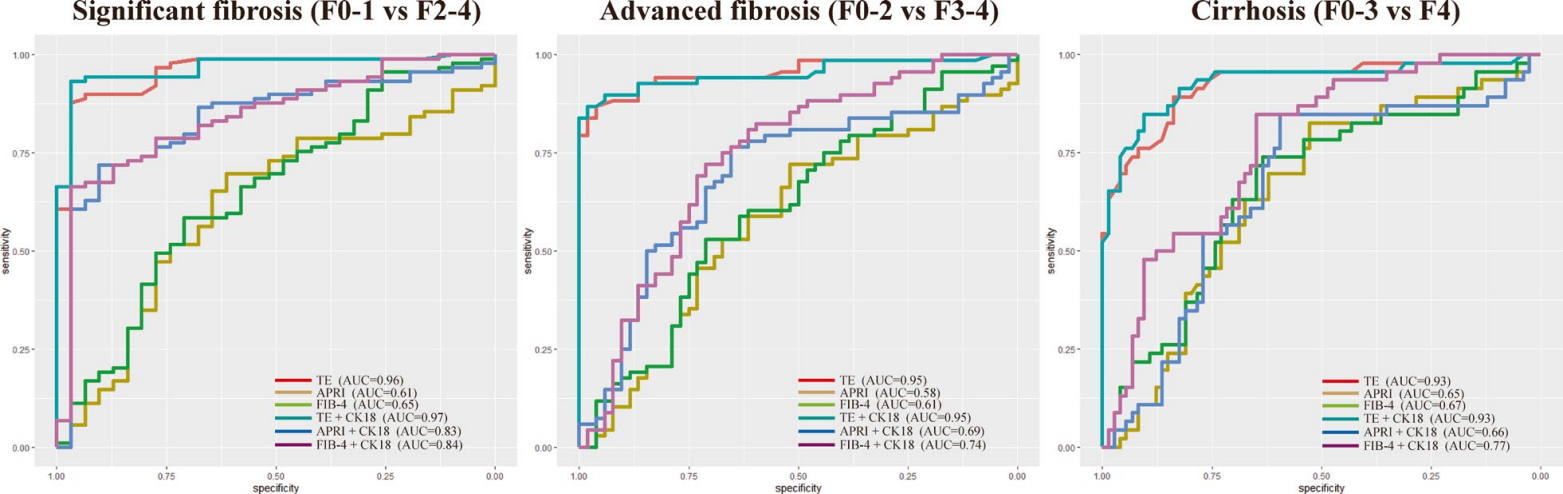
ROC curves of methods for predicting liver fibrosis in the validation cohort. APRI, aspartate transaminase-to-platelet ratio index; CK18, cytokeratin 18; FIB-4, fibrosis-4 index; ROC, receiver operating characteristic; TE, transient elastography

All ROC curves from the validation cohort are provided in Fig. 3. All AUC values and comparison results are summarized in Table 5. For predicting significant fibrosis, the observed AUC values of APRI + CK18 (AUC 0.83 [95% CI 0.75, 0.92]) and FIB-4 + CK18 (AUC 0.84 [95% CI 0.77, 0.92]) were higher than those of APRI (AUC 0.61 [95% CI 0.50, 0.73]; *P* < 0.001) and FIB-4 (AUC 0.65 [95% CI 0.53, 0.76]; *P* < 0.001). For predicting advanced fibrosis and cirrhosis, the AUC values of FIB-4 + CK18 (AUC 0.74 [95% CI 0.65, 0.83], 0.77 [95% CI 0.69, 0.86], respectively) were significantly higher than those of FIB-4 (AUC 0.61 [95% CI 0.51, 0.71], *P* = 0.02; 0.61 [95% CI 0.51, 0.71], *P* = 0.03). No significant differences between TE + CK18 and TE were observed.

**Table 5:** The AUROC values of prediction methods for significant fibrosis, advanced fibrosis and cirrhosis in the validation cohort

	Significant fibrosis (F0-1 vs F2-4)	Advanced fibrosis (F0-2 vs F3-4)	Cirrhosis (F0-3 vs F4)
TE	0.96 (0.92, 0.99)	0.95 (0.92, 0.99)	0.93 (0.87, 0.98)
APRI	0.61 (0.50, 0.73)	0.58 (0.47, 0.68)	0.65 (0.55, 0.75)
FIB-4	0.65 (0.53, 0.76)	0.61 (0.51, 0.71)	0.67 (0.57, 0.77)
TE + CK18	0.97 (0.94, 0.99)	0.95 (0.91, 0.99)	0.93 (0.88, 0.98)
APRI + CK18	0.83 (0.75, 0.92)	0.69 (0.59, 0.79)	0.66 (0.56, 0.77)
FIB-4 + CK18	0.84 (0.77, 0.92)	0.74 (0.65, 0.83)	0.77 (0.69, 0.86)
Comparison of AUROC			
TE and TE + CK18	.32	.60	.40
APRI and APRI + CK18	< .001	.19	.89
FIB-4 and FIB-4 + CK18	< .001	.02	.03

Numbers in parentheses are the 95% confidence interval

AUROC, area under the receiver operating characteristic; APRI, aspartate transaminase-to-platelet ratio; CK18, cytokeratin 18; FIB-4, fibrosis-4 index; TE, transient elastography

**Fig. 4:**
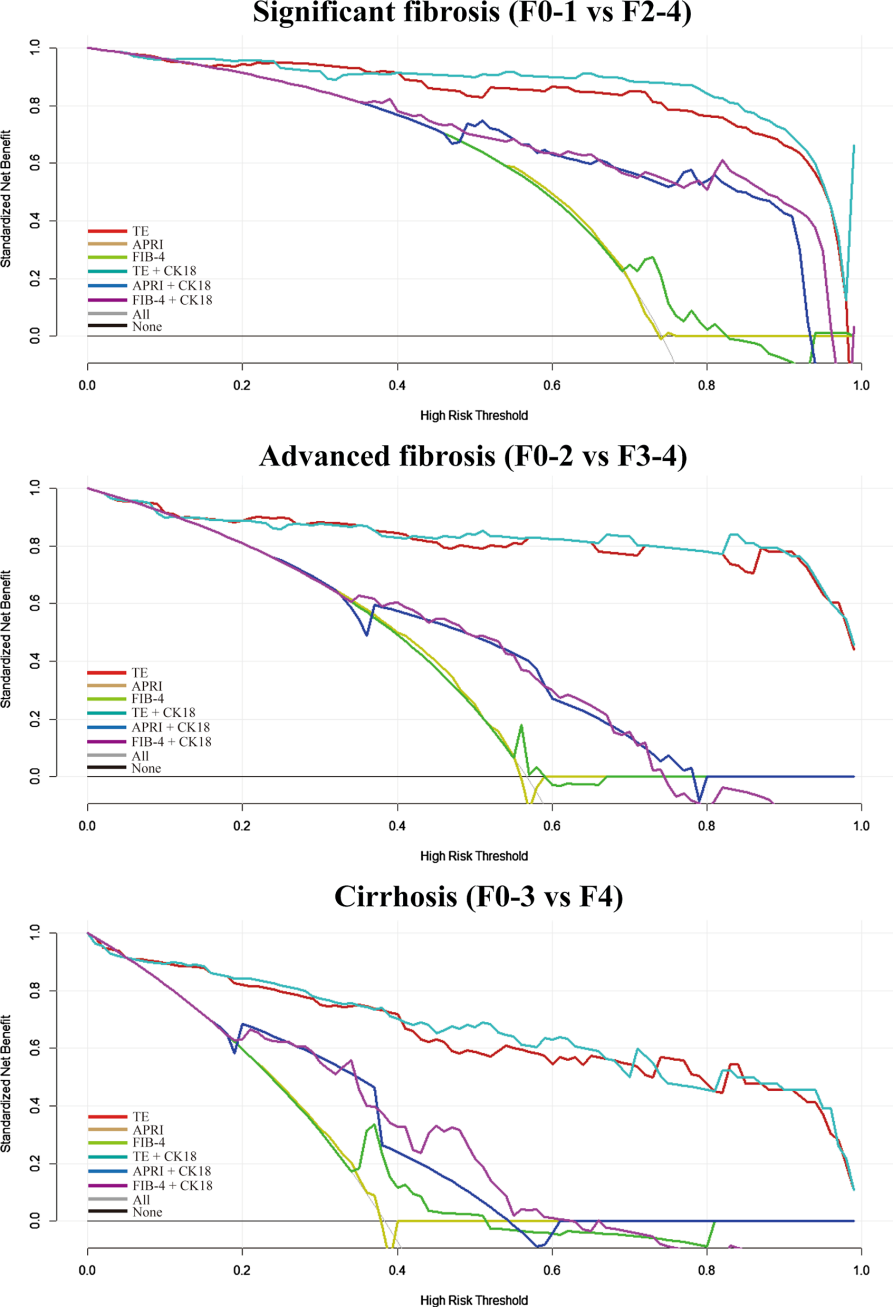
Decision curve analysis for each model in the validation dataset. The y-axis measures the net benefit. APRI, aspartate transaminase-to-platelet ratio index; CK18, cytokeratin 18; FIB-4, fibrosis-4 index; TE, transient elastography

The decision curve analysis for the diagnostic methods are presented in Fig. 4. Compared with scenarios in which no prediction model would be used (i.e. treat-all or treat-none scheme), the TE or TE + CK18 provides a better net benefit to predict fibrosis than the other models for threshold probabilities of more than 10%. Compared with isolated serum biomarker indices (APRI or FIB-4), APRI + CK18 or FIB-4 + CK18 provides more benefits for threshold probabilities of more than 40%.

## Discussion

In this study, we found the positive correlation between the level of CK18 and liver fibrosis stage (Spearman correlation analysis, *r* = 0.452, *P* < 0.001) in CHC patients with T2DM. CK18, TE and ALB were confirmed as independent predictors for liver fibrosis. In addition, combination Models that incorporate the previously proposed methods (APRI, FIB-4 and TE) and CK were established and validated for the prediction of significant fibrosis, advanced fibrosis and cirrhosis. Promisingly, the corresponding combination models of serum biomarker indices (APRI and FIB-4) showed better discrimination ability and more clinical benefits, thereby providing important information for medical decision support.

Our results demonstrated that CK18 has the predictive value for liver fibrosis in CHC patients with T2DM. For serum biomarker indices, CK18 can contribute to diagnosing the fibrosis stage. For liver stiffness measurement, CK18 cannot significantly improve the diagnostic performance due to enough favorable performance of TE [17, 18]. However, the Fibroscan device is still expensive and requires annual maintenance (€34,000 for a portable device and €5000 for its annual maintenance). In China, the machine is often accessible in the main hospitals [19]. Therefore, CK18 as an inexpensive alternative biomarker, can be combined with serum indices as inexpensive alternative methods for identifying patients with CHC and T2DM who need treatment.

CK18 is the predominant intermediate filament protein in the liver and contribute to substrates of caspases during hepatocyte apoptosis [20]. Levels of CK18 have been shown to be elevated in hepatocellular carcinoma, viral hepatitis, alcoholic hepatitis, NAFLD and cholestatic liver disease [21]. George et al. [22] reported that serum apoptotic caspase activity is associated with the severity of liver histologic lesions in both CHC and NAFLD.

In addition, HCV infection leads to a defect in insulin receptor substrate (IRS)-1 association with the IR and insulin signaling defects in hepatic IRS-1 tyrosine phosphorylation and phosphatidylinositol 3-kinase (PI3-kinase) association/activation, which contribute to insulin resistance [23]. Moreover, insulin resistance accompanied with the type 2 diabetes mellitus is positively associated with hepatic steatosis, causing an increased risk of liver fibrosis [24]. In this study, although the significant correlations between IR and CK18 (*r* = 0.160, *P* = 0.025) or liver fibrosis (*r* = 0.110, *P* = 0.026) were observed through correlation analysis, HOMA-IR is not confirmed as an independent predictor for liver fibrosis in CHC with T2DM. No significant correlation was found between CK18 and histologic liver steatosis. These might indicate that the insulin resistance might partly account for (not via liver steatosis) the correlation between CK18 and liver fibrosis. A study conducted by Jazwinski et al. [25] also demonstrated the similar conclusion that CK18 in CHC is related to advanced fibrosis but not steatosis. CK18 might be a indicator not only for liver fibrosis but also for insulin resistance in CHC with T2DM, which requires more large sample-size studies to investigate.

Several limitations should be noted in this study. First, inherent selection biases cannot be avoided due to the retrospective nature of this study. For instance, patients with unclear or unsatisfied results of noninvasive tests would accept liver biopsy. Absence of control group is HCV patients without T2DM is also a major limitation. We cannot conclude that the predictive value of CK18 is only special for CHC with T2DM. Further study should add the control group for more analyses. Finally, the combination models were established and validated on the basis of data obtained from a single center. Multi-institutional studies are required for further validations.

In conclusion, CK18 is an independent predictor of liver fibrosis for CHC patients with T2DM. It can provide added values to noninvasive methods for diagnosing fibrosis and help clinical decision-making.

## Data Availability

The datasets analyzed during the current study are available from the corresponding authors on reasonable request.
